# Smoking cessation opportunities in severe mental illness (tobacco intensive motivational and estimate risk — TIMER—): study protocol for a randomized controlled trial

**DOI:** 10.1186/s13063-018-3139-9

**Published:** 2019-01-14

**Authors:** María José Jaén-Moreno, Nuria Feu, Justa Redondo-Écija, Francisco Javier Montiel, Cristina Gómez, Gloria I. del Pozo, Jose Ángel Alcalá, Luis Gutiérrez-Rojas, Vicente Balanzá-Martinez, Geli Marie Chauca, Laura Carrión, Maria Isabel Osuna, María Dolores Sánchez, Inmaculada Caro, Miriam Ayora, Francisca Valdivia, María Soledad López, Jose Manuel Poyato, Fernando Sarramea

**Affiliations:** 1 Instituto Maimónides de Investigación Biomédica de Córdoba (IMIBIC), Córdoba, Spain; 2 Departamento de Ciencias Sociosanitarias, Radiología y Medicina Física, Universidad de Córdoba, Córdoba, Spain; 3 Unidad de Gestión Clínica de Neumología, Hospital Universitario Reina Sofía, Córdoba, Spain; 4 Unidad de Drogas y Adicciones, Instituto Provincial de Bienestar Social, Diputación de Córdoba, Córdoba, Spain; 5 Unidad de Gestión Clínica de Salud Mental, Complejo Hospitalario de Jaén, Córdoba, Spain; 6 Unidad de Gestión Clínica de Salud Mental, Hospital Universitario Reina Sofía, Avenida Menéndez Pidal s/n 14014, Córdoba, Spain; 7 Grupo de Investigación Psiquiatría y Neurociencias (CTS-549), Instituto de Neurociencias, Universidad de Granada, Granada, Spain; 8 Área de Psiquiatría y Psicología Médica. Departamento de Medicina, Servicio de Psiquiatría, Universidad de Valencia, CIBERSAM, Hospital Universitario y Politécnico La Fe, Valencia, Spain; 9 Unidad de Gestión Clínica de Salud Mental, Hospital Infanta Margarita, Cabra, Spain; 10 Unidad de Gestión Clínica de Salud Mental, Hospital Universitario Virgen de la Victoria, Málaga, Spain; 11 Unidad de Psiquiatría del niño y del adolescente, Hospital Universitario Gregorio Marañón, Madrid, Spain; 12 Centro de Investigación Biomédica en Red de Salud Mental, CIBERSAM, 33006 Oviedo, Spain

**Keywords:** Schizophrenia, Bipolar disorder, Tobacco cessation, Chronic obstructive pulmonary disease, Screening, Mobile technology

## Abstract

**Background:**

There is an increased risk of premature death in people with severe mental illness (SMI). Respiratory disorders and cardiovascular disease are leading causes of increased mortality rates in these patients, and tobacco consumption remains the most preventable risk factor involved. Developing new tools to motivate patients towards cessation of smoking is a high priority. Information on the motivational value of giving the lung age and prevention opportunities is unknown in this high-risk population.

**Methods/design:**

This article describes in detail a protocol developed to evaluate an intensive motivational tool, based on the individual risks of pulmonary damage and prevention opportunities. It is designed as a randomized, 12-month, follow-up, multicenter study. A minimum of 204 smokers will be included, aged 40 years and older, all of whom are patients diagnosed with either schizophrenia or bipolar disorder (BD). Chronic obstructive pulmonary disease (COPD) will be evaluated using spirometry, and the diagnosis will then be validated by a pneumologist and the lung age estimated. Based on this value, a motivational message about prevention will be issued for the intervention group, which will be reinforced by individualized text messages over a period of 3 months.

The efficacy of the method and the pulmonary damage variables will be evaluated: smoking cessation at the end of follow-up will be confirmed by *cooximetry*, and the COPD diagnosis and the severity of the staging for disease will be assessed.

**Discussion:**

In the context of community care, screening and early detection of lung damage could potentially be used, together with mobile technology, in order to produce a prevention message, which may provide patients with SMI with a better chance of quitting smoking.

**Trial registration:**

ClinicalTrials.gov, ID: NCT03583203. Registered on 11 July 2018.

Trial status: recruitment.

## Background

People living with serious mental illness (SMI) reduces life expectancy by up to 20 years [1]. Tobacco consumption, which is almost three times higher in this group than in the general population, is the main preventable risk factor for mortality [2]: half the patients who are unable to quit will die of causes related to smoking [3].

Despite the fact that the comorbidity between SMI and smoking makes it even more difficult to quit [4], there is growing evidence for the safety and short-term efficacy of the available treatments [3, 5, 6]. Possible ways of raising long-term smoking cessation rates in SMI patients include: improving the pre-treatment preparation phase, combining the available treatments and increasing the length of the maintenance phase [7].

Getting a patient ready to change their addictive behavior requires maximum motivation levels. The decision to continue smoking or to try to change this habit depends on a number of factors, including the balance between the perceived benefits and the risks of continued smoking. This balance can be influenced by personalized messages about the health risks they run, which serve as a “motivational trigger” [8]. In the general population, the most common incentive for giving up smoking is linked to concerns about current or future health problems [9]. Much less is known about the factors influencing the decision to quit in people with SMI. People with schizophrenia [10] and bipolar disorder (BD) [11] could have a lower appreciation for the health risks associated with cigarette smoking. Tidey et al. [12] describe how psychiatric smokers—parallel to the rest—who intended to quit, rated the importance of negative health consequences higher than those who did not. Motivation to quit smoking in this population might increase if they are provided with more tailored information on the negative health consequences of smoking [10, 12].

Respiratory diseases, especially chronic obstructive pulmonary disease (COPD) and pneumonia, are the main causes of death in patients with schizophrenia and BD, alongside cardiovascular disease and cancer [2]. Despite the high rates of smoking addiction, a higher-risk consumption (starting younger, higher levels of nicotine dependence and more intense smoking) [13] and the impact this has on mortality rates, the early diagnosis of COPD is not currently catered for in the health care system and studies of the respiratory damage involved are very rare. Even so, in schizophrenia and other non-affective psychoses, the existence of impaired lung function of possible multifactorial origin and the increased likelihood of obstruction [14] have been reported and the risk of delaying the diagnosis has been stressed [14]. The Spanish Consensus Statement on Physical Health in Schizophrenia and BD mentions the possibility of an increased risk of COPD compared with the general population [15, 16], although there is limited information about BD and this is generally inconclusive due to its lack of statistical power [14].

COPD is the most common respiratory pathology in the general population, and smoking is its main cause in the Western countries [17]: it is estimated that half of those who smoke are likely to suffer from COPD. The most important changes in lung function occur in the early stages of the disease, even before the development of COPD [18]. The reduction in forced expiratory volume in 1 s (FEV1), when adjusted for age and level of consumption, is, per se, a high-risk marker of general mortality [19], cardiovascular mortality [20] and lung cancer [21]. The main health care challenge we face here is early diagnosis [22], and quitting smoking is the only step which is capable of slowing down its progress.

Spirometry is a safe, simple, non-invasive and cost-effective technique, and is the gold standard test in the study of pulmonary function and the early diagnosis of COPD [23]. It can be carried out in the primary care setting and there is a general consensus over the criteria for diagnosis and staging [24]; in smokers over 40 years old, it diagnoses between 21 and 25% of COPD in asymptomatic patients [25]. Although initially, when performed in parallel with other biomedical tests, the value of both test and results in increasing motivation in the general population to quit smoking proved inconclusive [26], renewed interest has been shown in this technique following the results of a clinical trial which informed smokers of the calculation of their “lung age” and achieved an increase in the cessation rate [8, 27]. However, to our knowledge, there is no evidence available of how effective this intervention may be in smokers with SMI.

In the general population, a simple recommendation by the physician can influence and increase the chances of giving up smoking in the long term [28]. In populations at higher risk, such as smokers with an SMI, the recommended intervention is the 5 A’s (Ask, Advice, Assess, Assist and Arrange) [7] – however, there is still a need for greater intensity in generating motivation [29]. Currently, mobile technology represents a cost-effective way of promoting health and preventing disease and the information it can convey about health can help to prompt changes in behavior. However, although interventions through text messages (SMS) are capable of generating extra motivation to quit smoking in the general population, so far there is no evidence in more vulnerable populations, including smokers with SMI [30].

The problem of smoking in this population requires new effective strategies to motivate the patient to attempt to quit and aim for the ultimate goal of complete abstinence. Although the impact of respiratory disease on the mortality rates of these patients is clear to see, the possibilities of an early diagnosis of the damage and its influence on motivating the patient to change are more problematic. Given these needs and the lack of evidence, the information based on individual health risks and on creating chances for prevention provides us with an opportunity to assess the situation. It is possible to implement the study of respiratory damage in an environment of community care for mental health, and SMS technology will help to make the message clearer and more forceful.

In a high-risk population, the probability of finding premature lung damage would be higher. Providing information on prevention opportunities will help the patient move forward through the quitting decision. We therefore propose the following objectives:

- To measure the level of undiagnosed lung damage in smokers with SMI
- To evaluate the effectiveness of an intensive anti-tobacco intervention, which offers individualized information on lung damage and possibilities of prevention, as a way of helping patients diagnosed with schizophrenia or BD to quit smoking

## Methods/design

### Study design

This is a multicenter, open, randomized and prospective study, with a 12-month follow-up period. Nine Mental Health Centers from the South of Spain, serving both rural and urban populations in the provinces of Córdoba, Jaén, Málaga and Granada, will take part in the study.

The study protocol (version 1: 11/2016) has been approved by the Ethics Committee of Reina Sofía Hospital in Córdoba, Spain, and informed consent will be obtained from each participating patient before recruitment. The http://ClinicalTrials.gov identifier was NCT03583203.

### Recruitment and randomization (see Fig. 1)

This will be a pioneer study in populations with SMI and, for this reason, reference figures from similar studies [31] have been used to calculate the sample size, adjusted to the characteristics and severity of the sample. Abstinence after 12 months was estimated at 3 and 15% in the control and experimental branches, respectively. A level of confidence of 95% and a power of 80% is assumed, which requires a minimum of 85 patients per branch, with an added 20% for possible losses during follow-up.

**Fig. 1:**
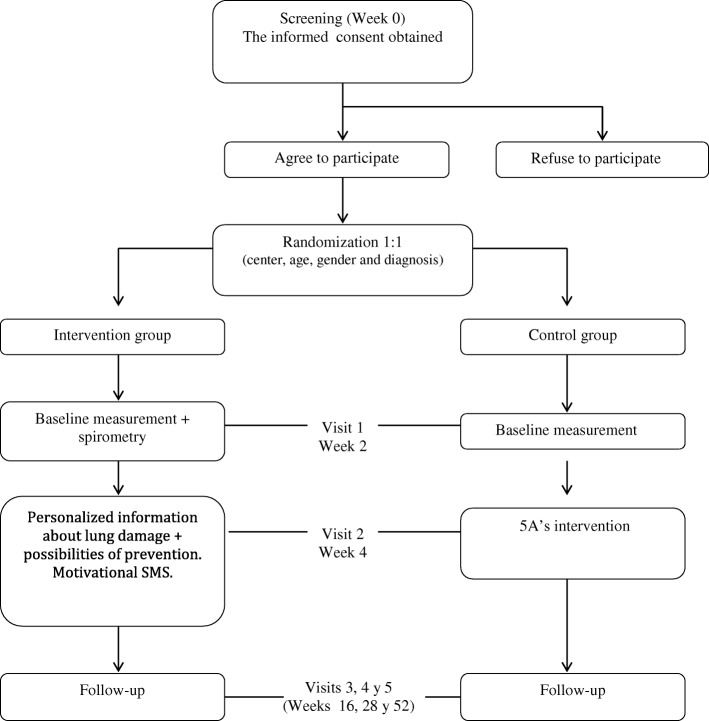
Flow diagram of study design

Recruitment of at least 204 patients who meet all the inclusion criteria and none of exclusion criteria will be carried out over 1 year and follow-up appointments will be arranged after that.

Inclusion criteria: (1) patients aged between 40 and 70 years; (2) confirmed diagnosis of BD or Schizophrenia according to *Diagnostic and Statistical Manual of Mental Disorders, 4th edition, text revision* (DSM-IV-TR); (3) active smokers who currently consume at least 10 CPD, with a cumulative consumption of 10 packets/year or more; (4) have signed an informed consent to participate in the study.

Exclusion criteria: (1) previous respiratory diagnosis of: asthma, cystic fibrosis, tuberculosis, simple chronic bronchitis, restrictive pulmonary disease or bronchiectasis; (2) acute respiratory symptoms; (3) heart disease or advanced oncological processes; (4) existence of a pathology which makes it advisable not to perform spirometry (recent pneumothorax, recent thoracic or abdominal surgery, aortic aneurysm, unstable angulation, retinal detachment, facial hemiparesis or oral/dental problems); (5) patients who, due to their intellectual disability or mental pathology, do not understand or cannot be forced to perform spirometry; (6) clinical instability with results of over 14 points on the Hamilton Depression Rating Scale (HDRS), a Young Mania Rating Scale (YMRS) score of over 6 or a Positive and Negative Syndrome Scale (PANSS) score of over 70.

The randomization will be carried out using the *Redcap* [32] program, as implemented by the Bioinformatics Unit of IMIBIC (the Maimónides Institute of Biomedical Research in Córdoba, Spain). It will be centralized, performed in permuted blocks and stratified by center, age, gender and pathology. The ratio will be 1:1 for each arm. The research staff will have no knowledge of the assignment until the patient signs their informed consent and has been included in the system (which will automatically assign the patient, once included, to a particular arm).

### Description of the intervention

The intervention will be performed by the regular health professionals who usually treat the patient (psychiatry and mental health nursing), after receiving specific training and after the intervention have been validated by specialists from the Addiction Network and the Pneumology Service at the Reina Sofía Hospital in Córdoba, Spain.

#### Control group

The control intervention lasts 30 min and will be structured around the 5 A’s technique (Ask, Advice, Assess, Assist and Arrange) [27]. Data will be collected on the patient’s smoking record, level of consumption and their individual level of motivation. Their smoking habit, personal circumstances and the general health risks derived from their current consumption will be discussed with the patient following the recommendations of the Treating Tobacco Guideline [33] and on the basis of the Motivational Interviewing [34]. The risks and benefits will be weighed and the patient’s perceived self-efficacy will be assessed in order to generate greater motivation. Depending on the degree of motivation, the idea of setting a deadline for the change in habit, specific treatment and regular follow-up will be put forward. In order to reinforce the message, informative materials stressing the general health benefits of quitting smoking will be provided, and patients will be given a direct contact number to request an urgent appointment for support and specific treatment, as needed.

#### Experimental group

This group will go through a spirometry study for a lung age and obstruction degree determination. The anti-smoking intervention will follow the same duration and general structure of the control group. In this case, when talking about the health risks, personalized information about the lung damage and prevention opportunities will be included. It will teach the patients about comparing lung age with chronological age and explain how the formula is calculated and the origin of the damage. After evaluating their COPD, the patients will be informed about its existence and staging. Depending on the damage found, the generation of motivation will focus on different prevention methods. Likewise, after the motivation level is set, the patients will be offered the option of treatment and regular follow-up. The intervention will be strengthened by motivational messages (designed by specialists from our Addiction Network) in an attempt to give continuity to personal message based on MI—half of which are linked to the possibility of preventing respiratory damage—and sent to the patient's mobile phone via SMS during the 3 months after the face-to-face intervention, with a decreasing rate: three messages per week in the first month, and two and one per week in the following months, respectively (see Table 1 for examples of these messages) [35]. Patients without mobile phones will receive a call on their phone to convey the same messages.

**Table 1:** Examples of text messages (SMS) sent to patients in the intervention group

Wanting to smoke is temporary, but the damage to your lungs is permanent – Which kind of suffering do you want to choose?	
The pleasure of breathing in deeply and enjoying a beautiful spring morning would be easier with cleaner lungs. How about trying it?	
Did that little run down the street leave you breathless? Ever thought about giving up smoking? Life is much more bearable with clean lungs.	
If your lungs could speak, they’d be really angry at how you are treating them. It’s up to you – you can make your life better.	
Better health and lung capacity, more cash to spend and less smelly clothes. That’s what you gain when you give up smoking. Difficult to imagine giving up? You’re not alone! Come and see us and we’ll plan it together.	

### Safety protocol

1. The anti-tobacco intervention can only be given to clinically stabilized patients. In these cases, their psychiatrist will confirm whether they can be classified as psychopathologically stable prior to the intervention scheduled for the second visit
2. Information about individual lung damage will be offered based on the prevention opportunities and following the general MI model, as in the rest of the protocol. To guarantee it, the messages will be generated based on respiratory variables and by the team of researchers— psychiatrist, expert physicians on addiction and pneumologist specialists—and sent on the Redcap platform to the center researcher
3. When they are first informed about the intervention and prior to the start of the intensive mobile phone intervention with SMS, the patients are asked once again if they wish to continue with the study. In each message sent, a phone number is included where the patient can request not to receive any more messages if they so wish
4. The patient is given the opportunity to contact the specialized addiction service network, made up of physicians who routinely treat patients with SMI who are trying to quit smoking and do not receive treatment due to their possible lack of psychopathological stability
5. The researcher will make a written record of any COPD diagnosis performed, which, in mild cases, is sent to the primary care physician and in moderate or severe cases, to the pneumology service

### Patient and public involvement

All the patients will receive enough information about the study by a resume paper with the principal objectives and methodology of the research. As was explained in Fig. 1, only the patients that give their agreement to participate will be included in the study. If the patients want, they will give us their contact details to receive the principal results published in the future.

### Result variables

1. Lung damage variables
  2.1 Main variable:
    Presence of COPD (FEV1/forced vital capacity (FVC) < 0.7, confirmed after bronchodilation) and staging (FEV1 > 80% – mild, FEV1 50–80% – moderate, FEV1 30–49% – severe)
  2.2 Secondary variable:
    Percentage of FEV1 compared with expected level. Calculation of lung age based on the Fletcher and Peto [36] model
2. Efficiency variables
  2.1 Main variable:
    Smoking cessation, defined as self-reported abstinence over the previous 7 days, confirmed by *cooximetry* with expired carbon monoxide (CO) < 10 particles per million (ppm)
  2.2 Secondary variables:
    Increased levels of motivation
    Number of attempts to quit and total duration in days of abstinence, as reported by the patient
    Smoking cessation objectified by cooximetry (< 10 ppm) at scheduled follow-up appointments
    Reduction in the number of cigarettes/day (CPD) smoked, as reported by the patient and confirmed by expired CO after 3, 6 and 12 months

### Data collection and monitoring (see Fig. 2)

1. Sociodemographic data:
  Age, gender, educational level, marital status, number of children, employment status, place of residence, home environment (including other members of the household who smoke) and income
2. Clinical variables:
  Confirmation of diagnosis. Schizophrenia and BD sections of the Spanish version of the Structured Clinical Interview for DSM-IV Axis I Disorders (SCID-I) [37]
  Psychiatric disease variables, as reported by the patient and confirmed by their clinical history. Age of onset, illness duration, number of suicide attempts and number of hospital admissions in the last 5 years
  Clinical stability will be assessed by the reference psychiatrist at each center and evaluated by the HDRS [38] and YMRS [39] scales for BD and PANSS [40] for schizophrenia
  Anxiety symptoms of anxiety. Hamilton Anxiety Rating Scale (HARS) [41]
  Global functioning. Global Activity Assessment Scale (GAAS) [42]
  Psychopharmacological treatment at study entry and during the study
3. Comorbidity (from medical history). Anthropometric measurements and vital signs (by nurse, following the schedule):
  Hypertension, diabetes, dyslipidemia, peripheral vascular disease, heart disease and oncological disease
  Height, weight, Body Mass Index (BMI), abdominal perimeter, oxygen saturation, blood pressure and heart rate
4. Smoking habit
  History of consumption: age of onset, years of consumption and accumulated consumption (number of packs/year)
  Current consumption and dependency level:
    Type and brand of tobacco and CPD
    Expired CO, reported as ppm, measured with an MD Diagnostic CO Check+ cooximeter (concentration range 0–99 ppm, sensitivity 0.1 ppm). The measurements will be obtained between 11.00 a.m. and 01.00 p.m., with a cut-off point for active smokers of 10 ppm, as in previous references [43].
    Dependence on nicotine, as evaluated by the Fagerström Test for Nicotine Dependence (FTND) [44]: mild (0–3), moderate (4–7) and severe (8–10)
  History of previous attempts to quit smoking (minimum 24 h trying to quit):
    Record of the number of attempts lasting more or less than 1 month. Total sum of days of abstinence reported
    Previous specific treatments, adverse reactions or psychopathological decompensation
  Motivation to quit smoking:
    Richmond Test [45]: low (0–4), moderate (5–6) and high (7–10)
    Analogue visual scale from 1 to 10, measuring the patient’s desire to quit smoking and perceived self-capacity [46]
    Prochaska and DiClemente’s Stages of Change (SOC) [47]
  Request for referral to specific treatment of Addictions Network:
    Number of appointments
    Treatment offered
5. Use of other substances
  The consumption of other substances—such as alcohol, cannabis, cocaine, stimulants, hallucinogens, heroin, methadone, other opiates or inhalants—will be measured using the “Drug Use Table: Individual Substances” section of the Spanish Addiction Severity Index, 6th version (ASI6) [48]
  We will record the age of the first consumption, years of regular consumption, consumption for 50 or more days in the patient’s life and consumption over the last 30 days. Consumption will also be calculated for caffeine, measured as cups of coffee/day (one cup of coffee = one energy drink = two cups of tea = three cups of soft drinks [49]), and for cannabis, measured as type (resin or leaf), consumption method (joint or vapor inhalation) and number of joints/year (52 weeks × number of joints/week/365 × number of years smoking) [50]. Patients with cocaine or heroin habits will be noted if the substance is inhaled
6. Physical activity
  The amount of physical activity will be calculated through the self-administered International Physical Activity Questionnaire (IPAQ), used in its shortened version [51] and validated in Spanish [52]. The unit to measure energy expenditure is the MET (Metabolic Equivalent of Task), and the total expenditure in METs-minute/week is calculated
7. Neuropsychological evaluation
  In order to evaluate neurocognitive functioning and any possible changes after the intervention, the Digit Span Test will be used in reverse order or backwards (WAIS-III) [53], together with parts A and B of the Trail Making Test [54], which measure verbal working memory, processing speed and executive attention, respectively

**Fig. 2:**
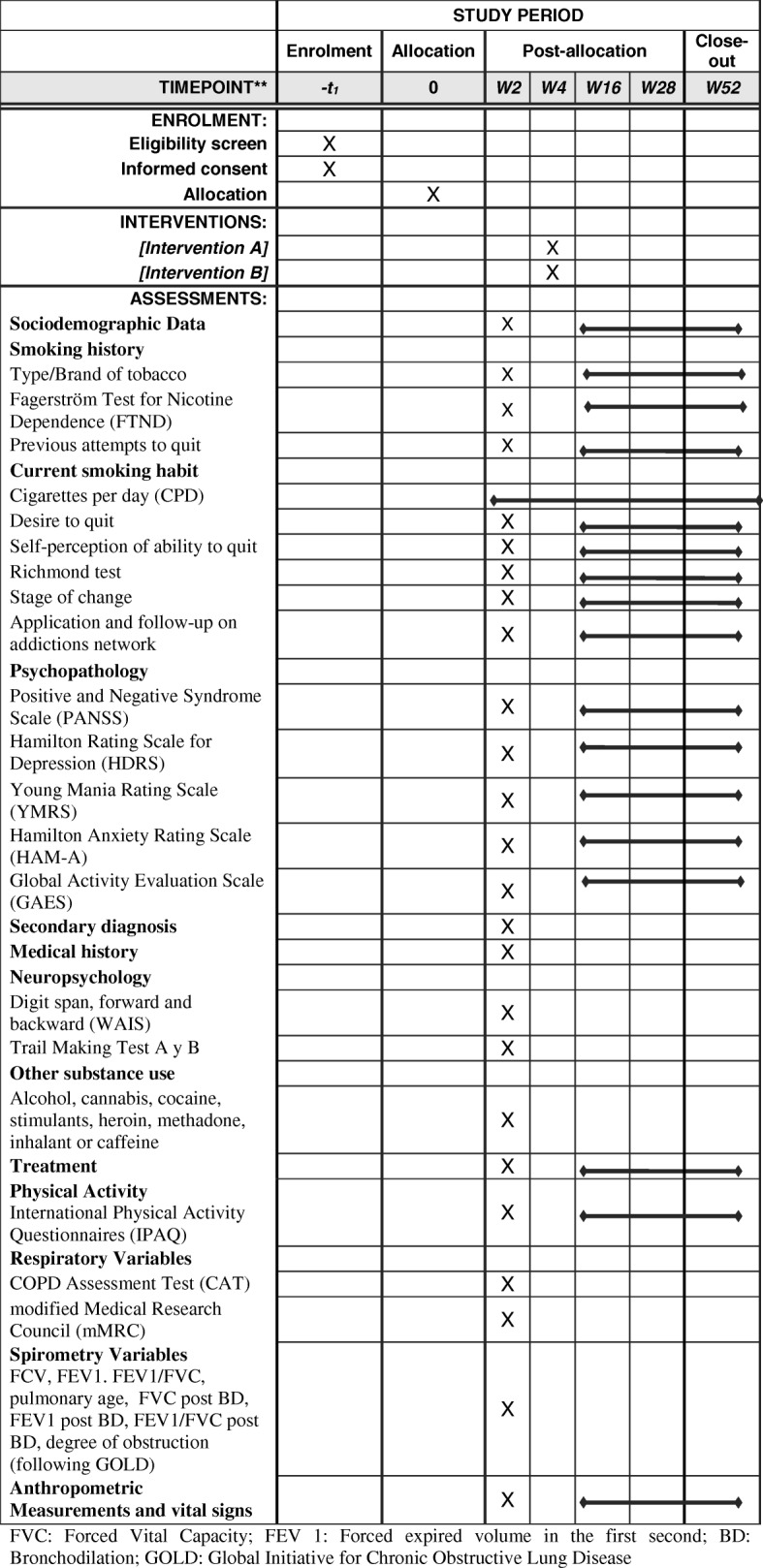
Scheduled tasks

### Spirometry

To validate the equipment used and check its characteristics, the test and criteria for quality control will be used for acceptability and repeatability of the results; the standardization criteria set down by the American Thoracic Society and the European Respiratory Society (ATS/ERS) [55] will be followed (see Table 2).

**Table 2:** Protocol for evaluation and diagnosis of respiratory pathology

Evaluation and validation	Persons responsible and venue	Nursing team (Mental Health Unit)
	Training	Accredited training^a^ given by the pulmonology service of Reina Sofía Hospital, Córdoba, Spain
	Skills acquired	Calibration, preparing patients, performing maneuvers, bronchodilation and repetition of spirometry
	Automatic Validation	Automatic validation Maximum of 8 maneuvers before achieving a minimum of 3 maneuvers of an acceptable standard^b^ Classification and automatic choice of the 2 best curves with repeatability criteria^c^
	Reversibility test	Repetition of 3 acceptable maneuvers 15 min after inhalation by bronchodilator (salbutamol, 400 μg)
Quantification and diagnosis	External Validation	External validation curves and volumes are assessed by a single researcher, head of the functional test unit of the Pneumology Service at the Reina Sofía Hospital, Córdoba, Spain
	Volumes	Forced vital capacity (FVC), forced expiratory volume (FEV1) and the quotient of the two (FEV1/FVC)
	Calculation of lung age	Automatic and externally verified
	Diagnosis and staging	Presence and degree of respiratory obstruction, according to criteria established by the Global Initiative for Chronic Obstructive Lung Disease (GOLD) guideline

^a^The accredited training consisted of learning the theoretical principles behind the test, how to handle and look after the material and how to perform spirometry maneuvers and secure the selected measurements

^b^Acceptability criteria: the flow/volume curve must not contain artifacts, must start well and should last for at least 6 s

^c^Repeatability criteria: the two highest FVC and the two highest FEV1, with a difference between them of less than 0.15 L

### Respiratory variables

- Respiratory symptoms and impact on quality of life [56]
- Severity of dyspnea, using the Modified British Medical Research Council (mMRC) scale [57]

### Statistical analysis

The statistical analysis will include all the patients randomized to both branches according to an intention-to-treat approach. The main variable for smoking cessation will be analyzed using the chi-square test for qualitative variables, which in this case consist of the differences between the control and intervention groups. The effect size will be expressed as relative risk (RR), absolute risk reduction (ARR) and the number needed to treat (NNT), with the corresponding 95% confidence interval (CI). The secondary variables will require the comparison of the average values of the quantitative variables between groups (level of motivation, consumption of tobacco according to CPD and expired CO) and will be carried out using Student’s *T* test and its respective post-hoc correction tests. The Mann-Whitney *U* test will be used for the non-parametric equivalent. The association between the quantitative variables will be evaluated using bivariate correlation tests, such as Pearson’s linear correlation coefficients (parametric test) or Spearman’s correlation coefficients (non-parametric test). The respiratory variables for the presence of COPD and its staging will require a descriptive analysis and the results will be presented as frequencies and percentages. Logistic regression models will be used for the multivariate analysis. To control any effects in the comparison between groups caused by contact with the addiction treatment network or the neurocognitive evaluation, the variables associated with these events will be collected and the corresponding adjustments made in the analysis.

## Discussion

Motivating smokers with SMI to undertake specific treatments to quit smoking is certainly challenging, and even more so to increase their chances of success [58]. In this context, our study may present an opportunity to develop and evaluate new models of motivational intervention. It is based on estimation of individual health risks and possible ways of prevention, whereas mobile technology allows greater intensity of the intervention.

In SMI patients, it is clear that comorbid smoking is severe and that the risk of respiratory illness increases the mortality rate. Even so, although we know that early diagnosis is critical, so far there have been no clinical recommendations about screening for damage in any age group or level of tobacco consumption. The presence of certain symptoms (cough, coughing up mucus, limited physical activity), the nature of the patients themselves (who are less likely to visit their physician for non-psychopathological complaints), and the type of health care they receive (more focused on mental health and less on primary care) all hinder an early diagnosis of the problem. However, in a population made up of patients with schizophrenia or BD who smoke, assessing the degree of decline in lung function or level of obstruction can provide evidence which can lead to prevention measures to help control serious health threats.

In a population being seriously harmed by smoking tobacco, the advent of mobile technology as part of health care (m-health) gives us a great opportunity to provide motivation messages at any time and place. The full potential and safety of its use in treating patients with psychotic disorders have been increasingly recognized in recent years [59]. Even so, although the most basic tools, such as SMS, have become commonplace in the treatment of smoking in the general population [60], their possible use in disadvantaged populations such as smokers with mental illnesses is still relatively unexplored [61].

Neurocognitive functioning will determine how the patients receive and make use of this information. In BD and schizophrenia have been described deficits in attention/processing speed, working memory and executive functions. These are key elements to maintain goal-directed behavior in general and stopping nicotine use in particular. The study design may allow a better understanding of how these variables influence the possible use of tools providing medical information in these populations.

Nonetheless, considering their obvious limitations, the results should be analyzed carefully. Despite the open design of this study, due to the characteristics of the intervention under evaluation, the main variables of efficacy and damage can be measured objectively through the techniques of cooximetry and spirometry. The intensity of the experimental intervention can be measured by two factors: the motivational value of the individualized information and the importance of keeping up motivation in the future. The control intervention is less intense, although much greater than the usual option offered in this type of population. The intervention is not designed to propose any specific treatment, but to prepare the patient for starting treatment and for attending, when ready, the specialized Addictions Network. Once in the real world the type of treatment, level of adherence and results will all be assessed objectively.

Community mental health care constitutes the ideal setting for developing an intervention model based on prevention. A multidisciplinary team who are familiar with SMI patients and their health needs can offer them, through regular follow-up checks, a new way of approaching the problem of smoking through early diagnosis and motivation. Studying the lung function using spirometry is a valid way of doing this, as long as the health professionals are suitably trained, and the results are accurately validated and interpreted by a specialist.

## Abbreviations

ASI6: Spanish Addiction Severity Index, 6th version
ATS/ERS: American Thoracic Society and the European Respiratory Society
BD: Bipolar disorder
BMI: Body Mass Index
COPD: Chronic obstructive pulmonary disease
CPD: Cigarettes/day
FEV1: Forced expiratory volume in 1 s
FTND: Fagerström Test for Nicotine Dependence
GAAS: Global Activity Assessment Scale
HARS: Hamilton Anxiety Rating Scale
HDRS: Hamilton Depression Rating Scale
IPAQ: International Physical Activity Questionnaire
MET: Metabolic Equivalent of Task
mMRC: Modified British Medical Research Council scale
PANSS: Positive and Negative Syndrome Scale
ppm: Particles per million
SMI: Serious mental illness
SMS: Text messages
SOC: Stages of Change
YMRS: Young Mania Rating Scale
